# A Quasi-Randomized Controlled Trial of an Integrated Healthcare Model for Patients with Coronary Heart Disease

**DOI:** 10.31083/j.rcm2307234

**Published:** 2022-06-24

**Authors:** Guilan Cao, Man Xie, Yulan Xu, Jindin Huang, Jing Liang, Baoming Tao, Qiaoyuan Yan

**Affiliations:** ^1^Department of Cardiology, Union Hospital, Tongji Medical College, Huazhong University of Science and Technology, 430022 Wuhan, Hubei, China; ^2^Department of Nursing Administration, Jieyang People’s Hospital, 522000 Jieyang, Guangdong, China; ^3^Department of Nursing Administration, Union Hospital, Tongji Medical College, Huazhong University of Science and Technology, 430022 Wuhan, Hubei, China

**Keywords:** coronary heart disease, integrated health care, secondary care, treatment outcome, patient compliance, quality of life

## Abstract

**Background:**

An increasing number of coronary heart disease (CHD) patients with an aging population are demanding available and effective out-of-hospital continuous healthcare services. However, great efforts still need to be made to promote out-of-hospital healthcare services for better CHD secondary prevention. This study aims to evaluate the effectiveness of a hospital-community-family (HCF)-based integrated healthcare model on treatment outcomes, treatment compliance, and quality of life (QoL) in CHD patients.

**Methods:**

A quasi-randomized controlled trial was conducted at the Department of Cardiology, a tertiary A-level hospital, Wuhan, China from January 2018 to January 2020 in accordance with the Consolidated Standards of Reporting Trials guidelines. CHD patients were enrolled from the hospital and quasi-randomly assigned to either HCF-based integrated healthcare model services or conventional healthcare services. The treatment outcomes and QoL were observed at the 12-month follow-up. Treatment compliance was observed at the 1-month and 12-month follow-ups.

**Results:**

A total of 364 CHD patients were quasi-randomly assigned to either integrated healthcare model services (n = 190) or conventional healthcare services (n = 174). Treatment outcomes including relapse and readmission rate (22.6% vs 41.9%; relative risk [RR] = 0.54; 95% confidence interval [CI], 0.40–0.74; *p* = 0.0031), the occurrence of major cardiovascular events (19.5% vs 45.4%; RR = 0.43; 95% CI, 0.30–0.59; *p* = 0.0023), complication rate (19.5% vs 35.0%; RR = 0.56; 95% CI, 0.39–0.79; *p* = 0.0042), and the control rate of CHD risk factors (*p *< 0.05, average *p* = 0.009) at the 12-month follow-up in the intervention group were better than those of the control group. There was no significant difference in treatment compliance at the 1-month follow-up between groups (*p *> 0.05, average *p* = 0.872). Treatment compliance at the 12-month follow-up in the intervention group, including correct medication, reasonable diet, adherence to exercise, emotional control, self-monitoring, and regular re-examination, was higher than that of the control group (*p *< 0.05, average *p* = 0.007). No difference was found in the compliance with smoking cessation and alcohol restriction at the 12-month follow-up between groups (*p* = 0.043). QoL at the 12-month follow-up in the intervention group was better than that of the control group (86.31 ± 9.39 vs 73.02 ± 10.70, *p* = 0.0048).

**Conclusions:**

The integrated healthcare model effectively improves treatment outcomes, long-term treatment compliance, and QoL of patients, and could be implemented as a feasible strategy for CHD secondary prevention.

## 1. Introduction

Coronary heart disease (CHD) still poses a considerable threat to global health and remains the main cause of premature death worldwide [1]. In China, the morbidity and mortality rate of CHD has increased year by year with the aging of the social population and changes in lifestyles [2]. The occurrence of CHD is closely associated with hypertension, dyslipidaemia, diabetes, obesity, smoking and other risk factors [3]. Although the extensive development of percutaneous coronary intervention (PCI) has effectively reduced the mortality of CHD, it still cannot eliminate the risk factors and change the natural course of the disease progression, after which recurrence is prone to occur [4]. The previous evidences have shown that standardized secondary prevention of CHD can significantly slow the disease progression, reduce the occurrence of adverse cardiovascular events, and improve the prognosis [5, 6].

However, for a long time, the treatment and management of CHD patients has mainly focused on hospitalization with little follow-up in China [7]. The traditional hospital-based healthcare model mainly concentrates on the treatment in acute phase and in-hospital cardiac rehabilitation, which leads to difficulties in the implementation of CHD secondary prevention outside the hospital. Thereby bringing about the inadequate implementation of rehabilitation and recurrence prevention for CHD, accompanied with the lack of awareness both by patients and health professionals on the significance of long-term care after discharge [8, 9]. Ultimately, this result in unsatisfactory control of the risk factors with a gap in the guideline recommendations and further has a negative impact on the patient’s prognosis and quality of life (QoL) [9]. Therefore, with regard to the optimization of healthcare model for CHD secondary prevention, great attention should be given to the improvement on out-of-hospital continuous care. Although some hospitals have attempted to conduct out-of-hospital continuous care services in recent years, most of them are still at the preliminary stage [8]. Efforts therefore need to be made to promote out-of-hospital healthcare services for the better CHD secondary prevention.

The “Outline of China’s Health Development Plan (2011–2015)” [10] has pointed out that China would establish and improve an “hospital-community-family (HCF)-based” healthcare services system to improve the ability to provide long-term services to patients with chronic diseases. The integrated healthcare model involves the comprehensive and multicomponent healthcare services that could integrate the patient as the centre, the family as the unit, the community as the supporting platform, and the hospital as the base to provide technical guidance. This innovative healthcare model has been gradually developed for patients with different type of chronic diseases such as patients with cardiovascular disease in chronic phase [11], but is still on the stage of development. Consequently, this study focuses on the limitation of out-of-hospital healthcare services, thereby developing and evaluating the effectiveness of a HCF-based integrated healthcare model for CHD patients in a tertiary hospital. The tertiary hospital integrating medical treatment, teaching, scientific research, and community health care has been responsible for the construction and management of the medical treatment alliance of many primary hospitals and community health service centres in Wuhan, China for many years. Through cooperation with the community, an HCF-based healthcare model has been designed for secondary prevention management of CHD and integrated by the multicomponent healthcare interventions provided by the hospital, community and family. The aims of this study were to examine the effectiveness of the HCF-based integrated healthcare model on the treatment outcomes, treatment compliance, and QoL of CHD patients.

## 2. Materials and Methods

### 2.1 Design

A quasi-randomized controlled trial (quasi-RCT) was adopted to evaluate the effectiveness of the HCF-based integrated healthcare model on the treatment outcomes, treatment compliance, and QoL of CHD patients compared to conventional healthcare services, including routine in-hospital healthcare, discharge guidance, and follow-ups.

### 2.2 Study Setting and Procedure

The study was conducted at the Department of Cardiology, a tertiary A-level hospital, Wuhan, China, from January 2018 to January 2020. Two large-scale communities in Wuhan city were selected to recruit the participants via the nonrandomized sampling method by research coordinators. The eligible participants were recruited prospectively from the hospital and quasi-randomly assigned to two groups based on the community they lived. The random number table was adopted to randomly allocate two communities to determine which one was the intervention group. The participants who lived in one community received the intervention with the HCF-based integrated healthcare model services while those who lived in another community received the conventional healthcare services. The random sequence of two communities was generated with the random number table by research coordinators. The assignment sequence was sealed until the patient was enrolled and allocated to interventions. Meanwhile, research coordinators, data collectors, data analysts, participants, healthcare workers responsible for family cares for patients, and physicians and nurses who worked at the clinics in two targeted communities were blinded to treatment assignments. The intervention in each group was conducted over 12 consecutive months from when the patient was admitted to the hospital to the 12-month follow-up.

### 2.3 Study Sample

A total of 364 CHD patients living in the two targeted communities who received regular medical treatment at the Department of Cardiology, a tertiary A-level hospital, were enrolled from the hospital as the study participants. The eligible participants were quasi-randomly assigned to two groups. Patients in one community were chosen as the intervention group, while patients in the other community were chosen as the control group. The inclusion criteria were participants with (1) CHD diagnosed by coronary angiography (CAG) or multislice coronary computed tomography angiography (CTTA) and (2) stable disease without serious complications after receiving regular treatment. The exclusion criteria were patients with (1) acute episodes of cardiovascular events and (2) cognitive and mental disorders.

### 2.4 Interventions

#### 2.4.1 The Conventional Healthcare Services

The control intervention was routine treatment and nursing care during hospitalization and follow-ups after discharge. The control group received accurate diagnosis and treatment plans provided by the hospital according to the health conditions of the patients. Before the patient was discharged from the hospital, the responsible nurses provided detailed instructions on discharge. Then, a homemade health education manual produced was issued, which included information about the introduction of common diseases, self-care knowledge, medication record cards, monitoring record forms of blood pressure and blood glucose, diet and activity precautions, review schedules, expert outpatient schedules and registration methods. Within 1 month after discharge, a follow-up visit was conducted by a specialist nurse via telephone every week. If there were no discomfort symptoms, after 1 month, the patient was called back once a month. Regular outpatient visits were also conducted in the patients. The community services centre would also establish health information files for the patients and further provide basic prevention and treatment measures for chronic diseases. The patients were allowed to seek medical services from the hospital or community if needed.

#### 2.4.2 The HCF-Based Integrated Healthcare Model Services

The tested intervention was the HCF-based integrated health care model program. The intervention group received the implementation of the HCF-based model, which included routine in-hospital care as implemented in the control group. The intervention methods were adopted as follows:

The Establishment of a Hospital-Centre Multidisciplinary CHD Management Team

A hospital-centre multidisciplinary CHD management team was established, including 2 deputy chief physicians, 2 attending physicians, 6 supervisor nurses, 2 dietitians, 1 rehabilitation technician, 2 psychological counselors, and general medical staff and home health care workers from the communities. CHD clinics were opened in the community. Physicians from hospitals and communities take turns providing consultations at the clinic. A CHD patient club, a specialized WeChat (an instant messaging and calling app) online chat group, and a QQ (an instant messaging and calling app) online chatting group was established for synchronous information exchange. At the same time, an information network interactive app platform was designed for all discharged CHD patients who participated in the tracking administration with informed consent. Integrated healthcare model services were implemented, in which HCF dynamic tracking management merged hospital, community and family care into a whole, and health electronic file recordings, two-way referrals, and community online appointments were all available to be conducted.

The Technical Support Provided to the Medical Staff in the Communities

With the aim of ensuring the proper application and successful delivery of the HCF-based model, knowledge and skills training on the application of this healthcare model, CHD prevention and care was conducted for community healthcare staff. The training method included multimedia teaching, case analysis, group discussion, operation demonstration, and real-world practice. Training on the application of the HCF-based model, CHD prevention, treatment, and nursing care was mainly carried out by deputy chief physicians and supervisor nurses from the multidisciplinary CHD management team 2–3 times a month for 2 hours each time. Training on CHD prevention, rehabilitation, and psychological interventions was also conducted by a psychological counselor and a rehabilitation technician once a quarter. The training duration lasted 6 months, and the assessment was conducted once a month. According to the monthly assessment results, the training focus was continuously adjusted the next month. The medical staff in the communities were also required to go to the hospital to participate in the concentrated training 1–2 times per quarter. It included training on the clinical diagnosis and treatment of CHD through live learning, the workflow of the chest pain centre, emergency PCI treatment, CHD drug treatment, emergency rescue, and intensive care. The medical staff in the communities were also allowed to participate in ward round practice carried out by specialists, which lasted for 1 year.

The Content of HCF-Based Integrated Healthcare Model

*Hospital-Based Intervention*

During hospitalization, psychological nursing care, including psychological evaluations, behavior observations, psychological communication, etc. was provided for patients. At the time of admission, the Symptom Checklist-90 (SCL-90) [12], a widely used psychological evaluation scale, was used to conduct preliminary psychological screening for all patients. For high-risk patients, the psychological counselors would further conduct the psychological intervention (i.e., speech therapy, supportive psychotherapy, cognitive therapy, music therapy, group psychotherapy, progressive muscle relaxation) and re-evaluation monthly. The related psychological intervention was adjusted based on the re-evaluation. At the same time, diet management was given to the patients to ensure nutritional balance, where the nutritionist assessed the nutritional status, blood glucose and lipid index, dietary habits, and daily activity level of the patients. The nutritionist formulated personalized recipes based on the results and provided diet instructions.

When discharged from the hospital, the patients were given detailed discharge guidance and a contact manual from the nurses. The nurses were also responsible for filling in the patient’s hospitalization information on the chronic disease management app platform and contacting the community to provide the patient information. The nurses further invited the patients and their families to join the chronic disease management platform, CHD management online WeChat group and QQ group. It was convenient to receive or check the health education arrangements, health knowledge and courseware information, expert outpatient time, review appointment, and online consultation with the aid of these online platforms or chat groups. The follow-ups were conducted by the hospital nurses via telephone every week in the first month after discharge. If no discomfort symptoms, the follow-ups via telephone were conducted once a month.

*Community-Based Intervention*

After the patients were discharged from the hospital, the community nurses came to verify the discharged patient’s related information. The community nurses explained the community CHD management plan, invited patients to join the patient club, and distributed the patient club activity schedule, community-free clinic schedule and schedule of community health education lecture. The nurses in the community visited the patients every month. The patients were also required to go to the community medical centre for reexamination every month and to go to the outpatient clinic at the hospital for review every 3 months. The health lectures were conducted in the community every month, and the community free clinic was conducted once every 2 months. The medical staff from the CHD management team shared responsibility for the above work, most of which was undertaken by hospital nurses. The lecture content included CHD knowledge, drug treatment, lifestyle changes, home care methods, self-care, and emergency care. Free health education materials were distributed to the patients on site. Patient club activities were also held every month to encourage the patients to exchange experiences with each other and build their confidence in fighting diseases. At the same time, to enrich patients’ spiritual lives, social volunteers were recruited to accompany patients to play chess, walk, chat, or teach patients Tai Chi, square dance.

*Family-Based Intervention*

The main caregivers of the patient would act as home health care workers, who would be initially and proactively evolved in the family-based intervention in this model. They would be responsible for the patient’s home care, including supporting the patient’s adherence to treatment according to the medication, lifestyle, exercise, and diet instructions given by the medical staff and urging the patient to maintain healthy behavior. If patients or home care workers had any questions, they were allowed to communicate and consult with the medical staff online at any time in the WeChat or QQ group.

*HCF-Based Integrated Interventions*

**Fig. 1.**
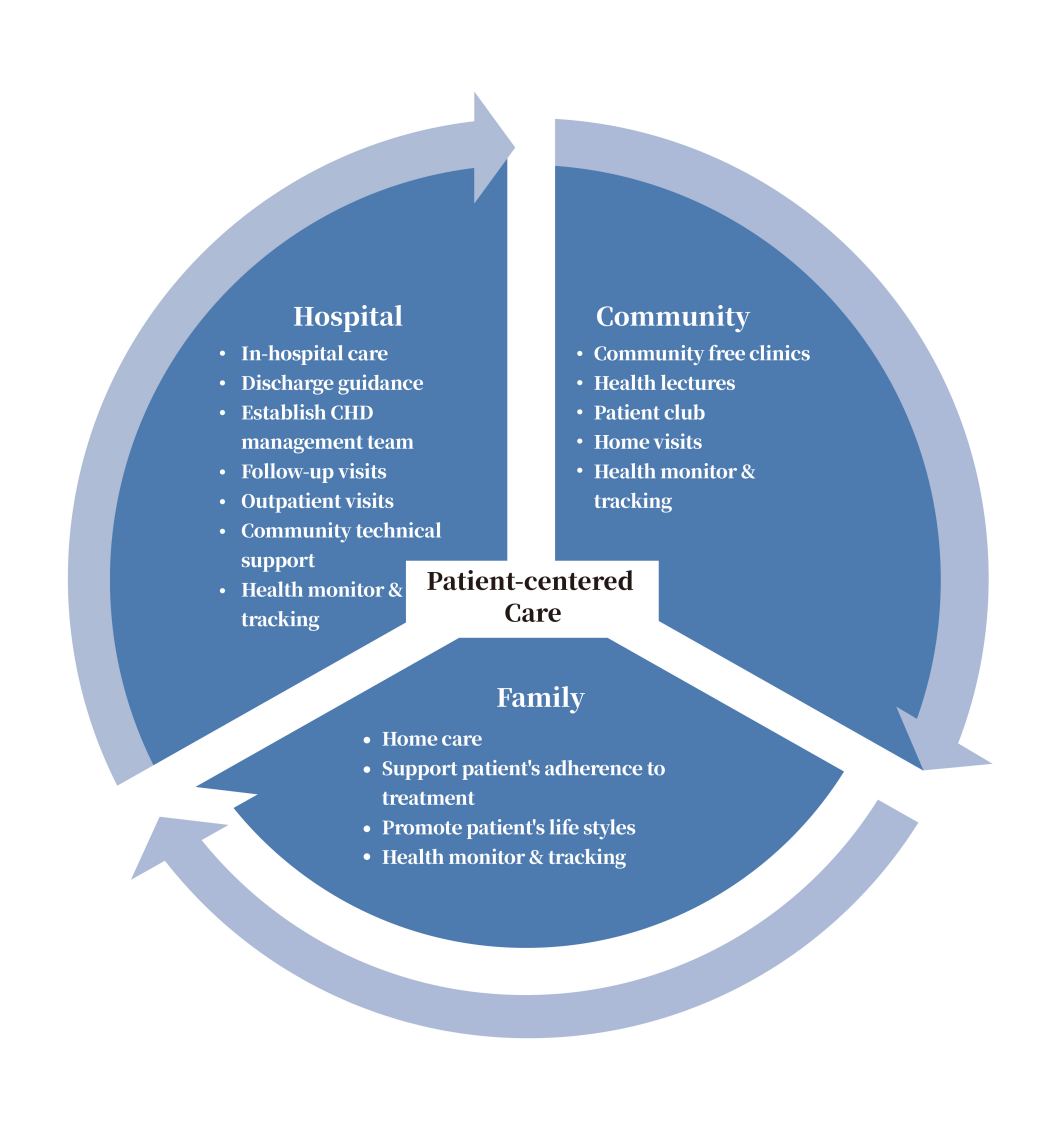
**HCF-based integrated healthcare model for CHD secondary prevention**.

The HCF dynamic tracking management system was established for patients in this model, which could achieve the function of health monitoring and tracking. The follow-up and review data, including the general data, all related data on the treatment outcomes and treatment compliance, and recordings on the outpatient visits, follow-up visits, and home visits, were all entered into the electronic health file in time. These allowed the physicians in the hospital and the communities to invoke it at any time. The patient was tracked and administered for 1 year. During this intervention period, the nurses reported the patient’s follow-up records to the home care workers every month to guide their work. Meanwhile, the establishment of a two-way referral platform module within the tracking management system made it available to carry out the two-way referral and community online appointment. Particularly for patients with poor treatment effects, the staff of the community contacted the hospital physicians, made an appointment online, and referred the patient to the hospital for ongoing treatment. Hospitalized patients with stable conditions could also be referred to the community for further rehabilitation. The HCF-based model is shown in Fig. 1. The comparison between the control and tested intervention is shown in Table 1.

**Table 1.** **The comparison between the control and tested intervention**.

Module	Description of measures	
	Intervention group	Control group
Hospital-based intervention	a. routine treatment and nursing care during hospitalization	a. routine treatment and nursing care during hospitalization
	b. detailed instructions on discharge	b. detailed instructions on discharge
	c. filling in the patient’s hospitalization information on the chronic disease management app platform	c. follow-up visits via telephone, every week in the 1st month after discharge and once a month after 1 month
	d. contacting the community to provide the patient information	d. regular outpatient visits after discharge, every 3 months
	e. inviting the patients and their families to join the chronic disease management platform, CHD management online WeChat and QQ group	
	f. follow-up visits via telephone, every week in the 1st month after discharge and once a month after 1 month	
	g. regular outpatient visits after discharge, every 3 months	
	h. establishing CHD management team	
	i. providing community technical support	
Community-based intervention	a. verifying the discharged patient’s home address, family situation and other related health information	a. establishing patient’s health information files
	b. explaining the community CHD management plan	b. providing basic prevention and treatment measures for chronic diseases if needed
	c. the health lectures in the community once a month, or in the community-free clinic every 2 months	
	d. patient club activities, every month	
	e. home visits, every month	
	f. community clinic follow-ups, every month	
Family-based intervention	a. the families or main caregivers act as home health care workers and take the responsibility for the patient’s all-round home care	a. the families or main caregivers act as home caregivers
	b. the health care workers communicate and consult with the medical staff online in the WeChat or QQ group if needed	b. the home caregivers consult with the medical staff at hospital outpatient department or community clinic if needed
HCF-based integrated interventions	a. establishing the HCF dynamic tracking management system	without a specific intervention
	b. regularly recording follow-ups and review data into the electronic health file	
	c. reporting the patient’s follow-up records to the home care workers monthly	
	d. health monitoring and tracking through the tracking management system	
	e. carrying out the two-way referral and community online appointment through a two-way referral platform module within the tracking management system	

### 2.5 Outcome Measures

#### 2.5.1 Primary Outcomes

The occurrence of readmission, which is one of the treatment outcomes of CHD, were considered as the primary outcomes in this study. The primary outcomes were measured by the questionnaire designed by the researcher. The questionnaire investigated whether the patient relapsed into the hospital.

#### 2.5.2 Secondary Outcomes

Treatment Outcomes

The other treatment outcomes of CHD included the occurrence of major cardiovascular events (angina pectoris, myocardial infarction, sudden death, restenosis, revascularization), the occurrence of complications (heart failure, arrhythmia, others), and the control rate of CHD risk factors (LDL-C, blood pressure, fasting blood glucose, glycated hemoglobin, body mass index). These outcomes were also measured by the questionnaire designed by the researcher. The questionnaire investigated whether there were new-onset complications or cardiovascular adverse events, and the control situation of associated risk factors through regular outpatient review, follow-up, and health file records on the HCF dynamic tracking management system. The reaching criteria of controlling risk factors associated with CHD mainly refers to the requirements of the 2017 AACE/ACE guidelines [13] (please see the **Supplementary Material**).

Treatment Compliance

A 7-component questionnaire designed by the researcher was adopted to assess treatment compliance (please see the **Supplementary Material**). The questionnaire consists of 14 items divided into 2 dimensions (medication management and lifestyle changes) and 7 components (correct medication, reasonable diet, quitting smoking & limit alcohol, exercise regularly, emotion management, self-monitoring, and regular follow-up). The questionnaire presents a total score ranging from 0 to 42 with a score of 0 to 3 for each item. A high score in each component represents high treatment compliance, within which 3 points represent complete compliance, 2 points refer to partial compliance, 1 point represents low compliance, and o points refer to noncompliance. A total score ≥18 points in the dimension of medication management (item 1 to item 7) was regarded as qualified compliance. A score of 3 points in each item of the dimension in lifestyle changes (item 8 to item 14) was regarded as qualified compliance. The questionnaire was evaluated by cardiologists from 4 class-A hospitals. The content validity of the questionnaire was 0.91, and the test-retest reliability had a kappa coefficient of 0.89.

QoL

QoL was evaluated by the Seattle Angina Pectoris Survey Scale (SAQ) for CHD [14]. The scale comprises a total of 5 factors and 19 items, which are the degree of physical activity limitation, angina pectoris stability, angina pectoris attack, treatment satisfaction, and subjective feelings of the disease. The total score of this scale is 100 points, which was divided into 5 levels. A higher score indicates a better patient’s body function status and quality of life. The scale has good test-retest reliability, content validity, structural validity and responsiveness [15].

### 2.6 Data Collection

The questionnaire of general data was designed by the researcher to collect the patients’ general information on admission, which included the patient’s age, gender, education, diagnosis, disease-related complications, economic status, and medical insurance. The data of outcome measures within the two groups of patients were measured and collected at baseline (before the intervention), at the 1-month follow-up, and at the 12-month follow-up. During the investigation, unified instruction was adopted to fully explain and ensure the understanding of patients. All questionnaires were completed by patients and retrieved after checking by investigators. The effective response rate before the intervention of the control group and intervention group was both 100%, while those after 1 month and 12 months were 86.57% and 93.60%, respectively.

### 2.7 Statistical Analysis

All data were input into the computer and analysed using SPSS, version 23.0 (SPSS Inc., USA). Categorical data including all primary outcomes were expressed as the incidence rate, and statistically significant differences were compared using the chi-square test. The measurement data with a normal distribution, including outcomes of treatment compliance and QoL, were expressed $\bar{X}$ using (±S), and the difference between groups was compared using the *t*-tests. Differences were considered statistically significant at *p *< 0.05. The 12-month data available were included in the per-protocol analysis, PP) as to evaluate the robustness of the primary estimates, while missing or unobserved data were excluded from the analysis.

## 3. Results

### 3.1 Recruitment, Attrition and Adherence

**Fig. 2.**
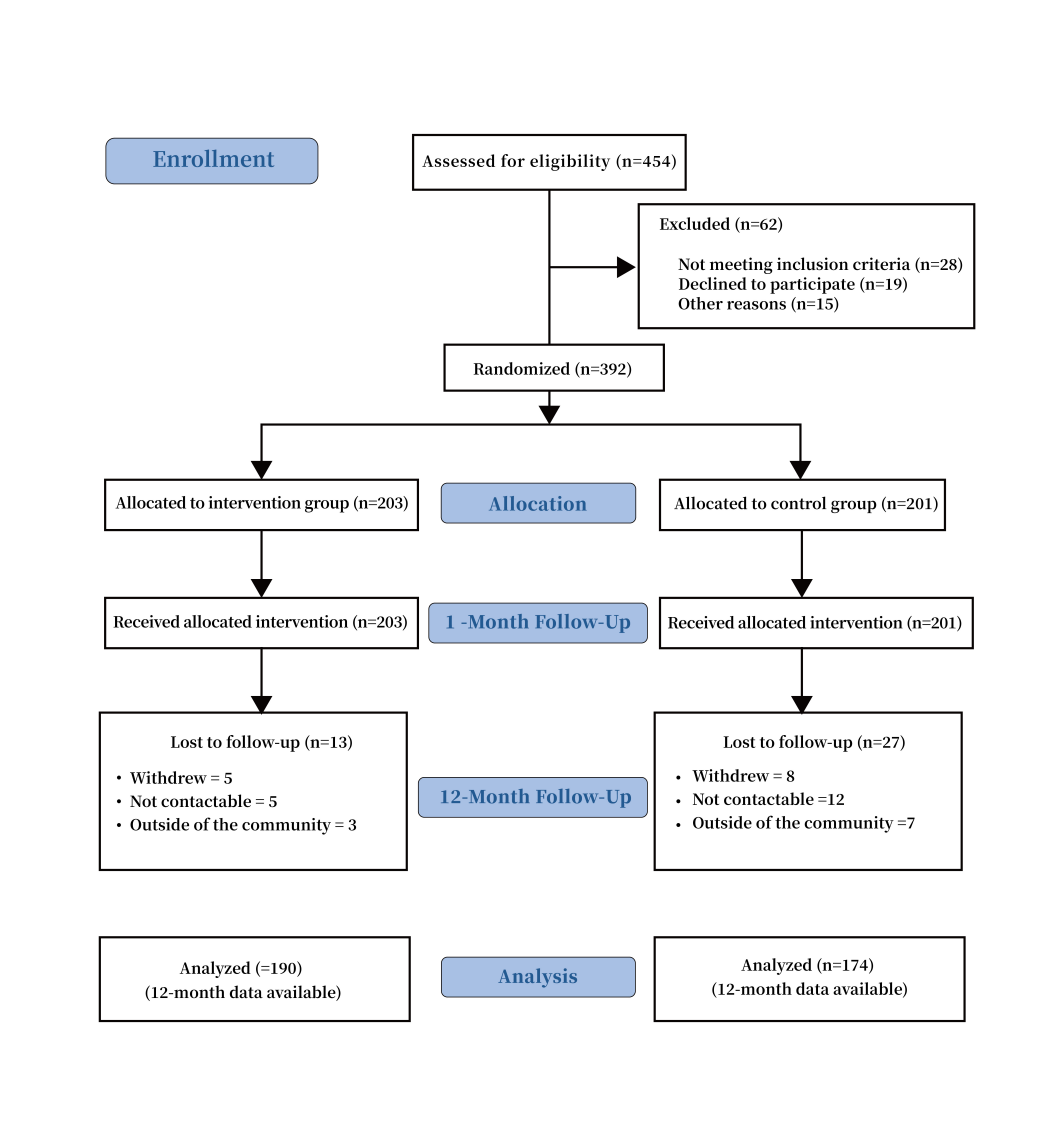
**The consort flow diagram of the process**.

The study ended when the study reached the planned sample size and length of follow-up goal. A total of 392 patients who met the eligibility criteria after screening consented to participate and were further enrolled in this study. Among them, a total of 203 participants were assigned to the intervention group, 13 of whom were lost to follow-up during the intervention process (attrition rate: 6.4%). A total of 190 participants were finally analyzed. Of 201 participants allocated to the control group, 27 were lost to follow-up (attrition rate: 13.4%). A total of 174 participants were finally analyzed. No adverse events occurred in this study. The Consort Flow Diagram of the Process is shown Fig. 2.

### 3.2 Participant’s Characteristic

There was no significant difference between the two groups in terms of demographic characteristics, including sex, age, education level, course of disease, severity of illness, comorbidities, economic conditions, and social support system (*p *> 0.05, *p* = 0.66). The baseline characteristics of the participants in the two groups were comparable, as shown in Table 2.

**Table 2.** **Baseline characteristics of study participants**.

		Intervention group (n = 190)	Control group (n = 174)	*$x^{2}$*/*t*	*p*
Gender, n (%)	Male	129 (67.9)	107 (61.5)	0.539	0.463
	Female	61 (32.1)	67 (38.5)		
Age, years, mean ± SD		65.39 ± 5.13	65.47 ± 5.22	1.201	0.065
Education, n (%)	Primary school or below	80 (42.0)	78 (42.4)	0.029	0.891
	High school or equal	46 (24.2)	41 (23.6)		
	College	33 (17.4)	31 (17.8)		
	Bachelor and above	31 (16.3)	29 (16.7)		
CHD type, n (%)	Angina type	93 (48.9)	86 (49.4)	0.331	0.583
	Myocardial infarction	62 (32.6)	50 (28.7)		
	Asymptomatic	24 (12.6)	25 (14.4)		
	Other	11 (5.8)	13 (7.5)		
Duration of CHD, year, mean ± SD	1∼	37 (19.5)	35 (20.1)	0.287	0.897
	5∼	75 (39.5)	69 (39.6)		
	10∼	55 (28.9)	51 (29.3)		
	15∼	23 (12.1)	19 (10.9)		
Complication, n (%)	No	93 (48.9)	87 (50.0)	0.271	0.792
	Heart failure	51 (26.8)	45 (25.8)		
	Arrhythmia	33 (17.4)	27 (15.5)		
	Other	13 (6.8)	15 (8.6)		
Financial burden, n (%)	Not at all	39 (20.5)	31 (17.8)	0.702	0.684
	Some	98 (51.6)	87 (50.0)		
	Very heavy burden	53 (27.9)	56 (32.2)		
Comorbidity, n (%)	Hypertension	117 (64.2)	49 (56.3)	1.317	0.358
	Diabetes	42 (47.3)	38 (43.6)	0.273	0.765
	Dyslipidaemia	73 (70.5)	57 (65.5)	0.627	0.561
	Over weight	61 (62.1)	51 (58.6)	0.358	0.692

### 3.3 Treatment Outcomes

Table 3 presents the treatment outcomes between the two groups over the 12-month intervention. The relapse and readmission rate (22.6% vs 41.9%; RR = 0.54; 95% CI, 0.40–0.74; *p* = 0.0031), major cardiovascular events (19.5% vs 45.4%; RR = 0.43; 95% CI, 0.30–0.59; *p* = 0.0023), and complications (19.5% vs 35.0%; RR = 0.56; 95% CI, 0.39–0.79; *p* = 0.0042) in the intervention group were significantly lower than those in the control group. The control rate of CHD risk factors in the intervention group were significantly higher than those in the control group (*p *< 0.05, average *p *= 0.009).

**Table 3.** **Treatment outcomes between groups at 12-month follow-up**.

	Intervention group	Control group	* $x^{2}$ *	*p*
	(n = 190)	(n = 174)		
Relapse and readmission rate, n (%)	43 (22.6)	73 (41.9)	8.917	0.0031
Major cardiovascular events, n (%)	37 (19.5)	79 (45.4)	12.625	0.0023
Angina, n (%)	15 (7.9)	26 (14.9)		
Myocardial infarction, n (%)	7 (3.7)	13 (7.5)		
Sudden death, n (%)	0	1 (0.6)		
Restenosis, n (%)	9 (4.7)	20 (11.5)		
Revascularization, n (%)	5 (2.6)	16 (9.2)		
Other, n (%)	1 (0.5)	3 (1.7)		
Complication rate, n (%)	37 (19.5)	61 (35.0)	9.106	0.0042
Heart failure, n (%)	11 (5.8)	21 (12.0)		
Arrhythmia, n (%)	20 (10.5)	35 (20.1)		
Others, n (%)	6 (3.2)	5 (2.9)		
LDL-C compliance, n (%)	161 (84.7)	81 (46.6)	26.249	0.0075
Blood pressure compliance, n (%)	163 (85.8)	89 (51.1)	23.895	0.0089
Fasting blood glucose, n (%)	148 (77.9)	91 (52.3)	15.673	0.0091
Glycated haemoglobin meets the standard, n (%)	155 (81.6)	81 (46.6)	24.679	0.0082
Body mass index compliance, n (%)	147 (77.4)	93 (53.4)	12.538	0.0097

### 3.4 Treatment Compliance

#### 3.4.1 Treatment Compliance Rate between Groups at 1-Month Follow-Up

At the 1-month follow-up, the treatment compliance rate of the two groups was more than 60%. After statistical testing, the difference was not significant (*p *> 0.05, average *p* = 0.872) (Table 4).

**Table 4.** **Treatment compliance rate between groups at 1-month follow-up**.

	Correct medication, n (%)	Reasonable diet, n (%)	Quit smoking & limit alcohol, n (%)	Exercise regularly, n (%)	Control emotion, n (%)	Self-monitoring, n (%)	Regular follow-up, n (%)
Intervention group (n = 190)	190 (100.0)	173 (91.0)	125 (65.8)	167 (87.9)	157 (82.6)	177 (93.2)	185 (97.4)
Control group (n = 174)	174 (100.0)	163 (93.7)	113 (64.9)	149 (85.6)	142 (81.6)	162 (93.1)	168 (96.6)
* $x^{2}$ *	–	0.037	0.032	0.072	0.077	0.029	0.382
*p*	–	0.873	0.861	0.835	0.806	0.859	1.000

#### 3.4.2 Treatment Compliance Rate between Groups at 12-Month Follow-Up 

12 months later, treatment compliance in the intervention group, including correct medication, reasonable diet, adherence to exercise, emotional control, self-monitoring, and regular re-examination, was higher than that in the control group (*p *< 0.01, average *p* = 0.007), and compliance with smoking cessation and alcohol restriction was higher than the control group (*p* = 0.043) (Table 5).

**Table 5.** **Treatment compliance rate between groups at 12-month follow-up**.

	Correct medication, n (%)	Reasonable diet, n (%)	Quit smoking & limit alcohol, n (%)	Exercise regularly, n (%)	Control emotion, n (%)	Self-monitoring, n (%)	Regular follow-up, n (%)
Intervention group (n = 190)	180 (94.7)	171 (90.0)	97 (51.0)	163 (85.8)	171 (90.0)	167 (87.9)	185 (97.4)
Control group (n = 174)	141 (81.0)	93 (53.4)	69 (39.7)	115 (66.0)	101 (58.0)	97 (55.7)	105 (60.3)
* $x^{2}$ *	9.537	31.727	3.739	11.964	26.715	22.729	37.427
*p*	0.002	0.0069	0.043	0.0092	0.0073	0.0082	0.0091

### 3.5 QoL

At the 12-month follow-up, the total QoL scores and scores of various factors in the intervention group were significantly higher than those in the control group (*p* = 0.0048), suggesting that QoL in the intervention group was better than that in the control group (Table 6).

**Table 6.** **Quality of life scores and related factors between groups at baseline and 12-month follow-up**.

	Baseline				12-month follow-up			
	Intervention group (n = 190)	Control group (n = 174)	*t*	*p*	Intervention group (n = 190)	Control group (n = 174)	*t*	*p*
Activity restriction, mean ± SD	71.72 ± 5.47	69.24 ± 7.19	1.713	0.082	87.39 ± 6.67	71.18 ± 10.51	8.546	0.0018
Angina pectoris, mean ± SD	65.38 ± 10.76	67.41 ± 12.12	–1.587	0.151	86.57 ± 10.47	72.29 ± 11.24	4.371	0.0079
Angina attacks, mean ± SD	70.48 ± 15.28	72.83 ± 14.86	–0.564	0.593	88.52 ± 10.73	75.42 ± 12.47	6.483	0.0043
Treatment satisfaction, mean ± SD	79.51 ± 15.37	78.69 ± 16.58	0.329	0.698	89.52 ± 7.74	80.73 ± 5.49	7.514	0.0035
Subjective feeling of disease, mean ± SD	71.64 ± 15.69	68.97 ± 16.73	–1.317	0.358	79.56 ± 11.34	65.47 ± 13.78	4.183	0.0081
Total score, mean ± SD	71.75 ± 12.51	71.43 ± 13.50	0.937	0.485	86.31 ± 9.39	73.02 ± 10.70	6.786	0.0048

## 4. Discussion

The occurrence, progression, and prognosis of CHD are closely associated with lifestyle, thereby requiring long-term medical care services and adherence to treatment [16]. However, with the extension of the discharge time, CHD patients generally suffer from poor long-term treatment compliance [17]. The decreased treatment compliance may occur due to economic factors, inconvenience in drug purchase, little out-of-hospital follow-up, insufficient health education, low families’ participation, etc. [18, 19]. Therefore, poor long-term treatment adherence to recovery after discharge of CHD patients is often found in the traditional hospital-based healthcare model. The results of this study showed that the treatment compliance rate of the two groups was more than 60% 1 month after discharge, and the difference between the two groups was not statistically significant. These are mainly related to the reality that the majority of patients have good adherence to the doctor’s instructions in the short term after discharge. However, the treatment compliance of the intervention group was significantly better than that of the control group 12 months after discharge. This suggests that the application of the HCF-based model could improve patients’ long-term treatment compliance, compared with the traditional hospital-based healthcare model. Similar results were found in the recent study, which also focuses on the positive effects of HCF linkage care on the long-term compliance of CHD patients [20]. The HCF-based model makes it possible to form a multidisciplinary management team for CHD, rationally allocate medical resources, and strengthen the HCF health care connection. The effective coordination and continuity of care from the in-hospital treatment to the discharge recovery in the community and family could be achieved in this integrated model [21]. This model allows us to carry out various types of follow-up activities, community health education, and comprehensive family supervision, together with real-time dynamic management of patient follow-ups. It could improve the accessibility and integrity of out-of-hospital care for CHD patients. Meanwhile, it could effectively utilize modern information platforms to interact with patients in real time, and provide ongoing reminders and surveillance given by the health professionals. The ongoing contact with CHD patients has been considered significant in maintaining lifestyle changes and healthy behaviors [20]. Therefore, the comprehensive strategies used in this model could further contribute to the better awareness of the disease and long-term treatment compliance among CHD patients.

The study results suggest that the HCF-based model could achieve better treatment outcomes, compared with the traditional hospital-based healthcare model. Bosselmannl *et al*. [21] suggested that comprehensive intervention with multiple risk factors and taking further preventive measures have also become new strategies to prevent the occurrence and delay the progression of the disease, which can reduce the morbidity and mortality of CHD. Studies also suggest that out-of-hospital interventions show positive effects on lifestyle changes in CHD patients [6, 20]. Moreover, home-based management can significantly reduce the CHD risk factors [22]. Similarly, the HCF-based model seems to be an effective strategy for CHD secondary prevention, which corporate comprehensive healthcare intervention from the hospital, community, and home. Previous studies have highlighted the situation of inadequate follow-ups and the urgent need for aggressive secondary prevention strategies to optimize long-term care for CHD patients [7, 8]. In this study, the implementation of the dedicated home visits, community and hospital follow-ups, periodic review of patients, and the personalized and targeted interventions according to the patient’s health conditions could be conducted continuously, which ensure the positive lifestyle intervention and effective control of risk factors of CHD patients. The recent clinical studies have also shown the positive effects of dedicated follow-ups on the improved treatment outcomes and the cardiovascular risk factor burden reduction for CHD patients [23, 24]. At the same time, the designed HCF dynamic tracking system implemented in this integrated model could be a useful tool to manage health and lifestyle. Data have shown that the decline in CHD mortality in developed countries is mainly due to the effective control of risk factors [25, 26]. Studies have also suggested active lifestyle intervention can effectively lower blood pressure, blood sugar, low-density lipoprotein cholesterol, and triglycerides, increase high-density lipoprotein cholesterol, and reduce patients’ cardiovascular risks [18, 27]. Consequently, the disease risk factors could be effectively controlled, thereby reducing the occurrence of disease recurrence, cardiovascular adverse events and complications, and improving patient outcomes in this innovative healthcare model.

The study results also showed that the QoL in the intervention group and the single scores of various factors were all markedly higher than those in the control group 12 months after the implementation. These illustrated that the application of the HCF-based model can effectively improve the long-term QoL of CHD patients. It is well known that effective hospital therapy and recovery after discharge both play a crucial role in the QoL of CHD patients [27]. The characteristics of the HCF-based integrated model are mainly to make full use of the professional technical advantages of tertiary hospitals. By providing professional technical guidance and training to community-level primary care workers, the ability to prevent and treat CHD in the community is improved, patient trust in community medical care technology is enhanced, and difficulties in seeking medical care are reduced. It is also highlighted that scientific community healthcare is of great clinical importance for maintaining health habits or behaviors, slowing the disease progression, and improving the QoL of CHD patients through regular healthcare intervention and effective follow-ups [28, 29]. At the same time, home-based care involved in this integrated model can also markedly reduce the disease risk factors and improve the QoL [21]. Moreover, the real-time reminder and concentration provided by home care workers to the patient could also improve the patient’s attention and sense of self-value. Meanwhile, the patient club can maintain the patient’s communication with the outside world, as well as give the patient a sense of belonging, which benefits the patient’s psychological balance, which is consistent with the study conducted by Bigdeli & Rahimian [30]. Additionally, the model tightly comprised the hospital, community, and family, together with the hospital-community real-time information exchange, two-way referral, and online appointment. This further helps to build an all-round protective circle for CHD treatment and rehabilitation, which enhances patient confidence and treatment enthusiasm, thereby forming a virtuous circle to improve the QoL.

## 5. Study Limitations

It should be noted that there are also some limitations in this study. First, there was a lack of a cost-effectiveness evaluation on the HCF-based integrated healthcare model in this study, which was also crucial for the sustainable translation of this model to practice for health services. Secondly, given that the evaluated intervention was a new complex healthcare model involving multicomponent interventions, the study was only conducted in two communities with a small sample size without complete randomization and sample size calculation. This may lead to the underpower of the accurate estimation of intervention effects. Meanwhile, attrition bias may also exist in this study with the regard that some study participants were withdrawn due to various reasons during the long period of follow-ups. In addition, the majority of outcomes in this study were self-reported, including treatment compliance and QoL, which may be susceptible to recall bias and inaccurately estimate problems. Nevertheless, to the best of our knowledge, this is one of the large controlled clinical trials conducted in two large communities to evaluate the effects of an innovative integrated healthcare model for CHD patients, which focused on better CHD secondary prevention. With regard to reduce the risk of bias and sample contamination effects, several solutions were adopted in this study. Firstly, the study recruited participants from two independent communities separated by large geographical distances and allocated the interventions at cluster levels of communities, which could mitigate contamination. Secondly, the random allocation of two communities, the allocation concealment, and blinding to research coordinators, data collectors, data analysts, participants, healthcare workers, and physicians and nurses in the community clinics was conducted strictly in this study, which may minimize the potential risk of selection bias, perform bias, detection bias, and contamination bias. Meantime, the structured intervention manual was provided during the training among the research teams to formalize the differences between interventions. The training meetings were also arranged to emphasize the importance of maintaining usual care for the control group and raise awareness of research teams and the involved medical staff on the importance of mitigating contamination. The clinicians and nurses were asked to sign a confidentiality agreement to state that they would not share the contents of the interventions between groups. Furthermore, the questionnaire or survey scale adopted in this study was found to have good validity and reliability in previous research. At the same time, each patient was given instructions on participating in the survey and required to write down the related outcomes in the daily routine. The solutions that have been adopted may mitigate recall bias and ensure the accurate estimate of outcome measures as possible. Therefore, the successful implementation of this study and the positive study findings could illustrate the feasibility and applicability of this integrated healthcare model, which appears to support the hypotheses of the potential positive effects of this integrated model on CHD secondary prevention. However, regarding the limitations of this study, future RCT with adequate randomized allocation, a robust sample size calculation, and a multi-center study design is further warranted to examine and confirm the effectiveness of this innovative integrated healthcare model.

## 6. Conclusions

Healthcare administrators and professionals should attach importance to the promotion of out-of-hospital continuous healthcare services for CHD secondary prevention. In this study, the HCF-based integrated healthcare model is beneficial to CHD patients in improving treatment outcomes, treatment compliance, and QoL, compared with the conventional hospital-based healthcare model. This integrated model could be implemented as a feasible strategy for CHD secondary prevention. Future research with a larger sample, more rigorous study design, and economic evaluation is recommended to further evaluate and confirm the effects and cost-effectiveness of this model.

## Abbreviations

HCF, hospital-community-family; CHD, coronary heart disease; QoL, quality of life; PCI, percutaneous coronary intervention; RCT, randomized controlled trial; CAG, coronary angiography; CTTA, coronary computed tomography angiography; SCL-90, symptom checklist-90; LDL-C, low-Density lipoprotein cholesterol; BP, blood pressure; FBG, fasting blood glucose; HbA1C, glycated hemoglobin; BMI, body mass index; AACE, American association of clinical endocrinologists; ACE, American college of endocrinology.

## Supplementary Material

Supplementary material associated with this article can be found, in the online version, at https://doi.org/10.31083/j.rcm2307234.
