# Supplementary material for: A Quasi-Randomized Controlled Trial of an Integrated Healthcare Model for Patients with Coronary Heart Disease
Source: Rev Cardiovasc Med. 2022 Jun 24;23(7):234. doi: 10.31083/j.rcm2307234 (PMC11266795; doi:10.31083/j.rcm2307234)
Supplement: Supplementary file 1 [file 2153-8174-23-7-234-s1.docx]

# Supplementary Material

Coronary Heart Disease Treatment Compliance Questionnaire

**Dear Mr./Ms:**

Thank you for participating in our survey, which aims to analyze your treatment compliance on medication management and lifestyle changes in the last month or year. Please answer all of the questions and tick each item with “√” in the tick boxes according to your daily routine in the past month or year. Please remember that your answers are confidential and that you should respond to each question as truthfully as possible.

|  | **Completely (3 points)** | **Frequently (2 points)** | **Occasionally (1 points)** | **Not at all (0 points)** |
| --- | --- | --- | --- | --- |
| 1. Do you take your medicine according to the type and dose of medicine prescribed by the doctor? |  |  |  |  |
| 2. Do you take your medicine according to the prescribed time? |  |  |  |  |
| 3. Do you take your medicine according to the prescribed frequency? |  |  |  |  |
| 4. Do you bring along your medicine with you when you travel or leave home for a long time? |  |  |  |  |
| 5. When you feel worse, you do not increase the medicine dosage by yourself. |  |  |  |  |
| 6. When you feel better, you do not reduced or stopped taking your medicine by yourself. |  |  |  |  |
| 7. You haven't missed your medication. |  |  |  |  |
| 8. Do you follow the dietary requirements on salt (< 5g/day) , cholesterol, trans-fats, and saturated fats limits? |  |  |  |  |
| 9. Do you follow the recommendations on quitting smoking and limiting alcohol? |  |  |  |  |
| 10. Do you follow the recommended exercise frequencies and times: 4-6 times weekly and at least 30 minutes for each time? |  |  |  |  |
| 11. Do you participate in recommended moderate exercises (e.g. walking, brisk walking, riding a stationary bike, swimming, Taiji, etc.) and avoid strenuous exercise? |  |  |  |  |
| 12. Can you control your emotions, maintain emotional stability，and avoid emotional excitement or tension? |  |  |  |  |
| 13. Can you regularly monitor your conditions by yourself , including blood pressure, blood glucose, heart rate, and symptoms? |  |  |  |  |
| 14. Do you have regular visits followed by the doctor’s advice (once /1-3 months)? |  |  |  |  |

Supplementary Table 1. Summary of Reasons for the Non-eligibility of the Participants in the Study.

| Reasons for non-eligibility | Participants: n(%) |
| --- | --- |
| Declined to participate | 19 (30.6) |
| Acute episodes of cardiovascular events | 18 (29.0) |
| Cognitive disorders | 5 (8.1) |
| With severe complications | 5 (8.1) |
| Disease condition deteriorated before consent | 9 (14.5) |
| Inconvenience to cooperate with the follow-ups | 6(9.7) |

Supplementary Table 2. The Treatment Goals of Controlling Risk Factors Associated with CHD.

| **Parameters** | **Risk Factors** | **Treatment Goals** |
| --- | --- | --- |
| LDC-C | High LDC-C | <55 mg/dL for risk category at extreme risk as described below:   - Progressive ASCVD including unstable angina in patients - after achieving an LDL-C <70 mg/dL - Established clinical cardiovascular disease in patients with - DM, CKD 3/4, or HeFH - History of premature ASCVD (<55 male, <65 female)   <70 mg/dL for risk category at very high risk as described below:   - Established or recent hospitalization for ACS, coronary, carotid or peripheral vascular disease, 10-year risk of a coronary event determined by Framingham risk scoring^a^ >20% - Diabetes or CKD 3/4 with 1 or more risk factor(s) - HeFH |
| BP | Hypertension | < 140/90mmHg for age under 65 years  < 150/90mmHg for age over 65 years |
| FBG | Diabetes mellitus or dysglycemia (impaired fasting glucose and/or impaired glucose tolerance) | 4.4-7.0 mmol/L |
| HbA1C | Diabetes mellitus or dysglycemia (impaired fasting glucose and/or impaired glucose tolerance) | < 7% |
| BMI | Overweight or obesity | < 24 kg/m^2^ |

Abbreviations: CHD, coronary heart disease; ASCVD, atherosclerotic cardiovascular disease; DM, diabetes mellitus; CKD, chronic kidney disease; HeFH, heterozygous familial hypercholesterolemia; ACS, acute coronary syndrome; LDC-C, low-density lipoprotein cholesterol; BP, blood pressure; FBG, fasting blood glucose; HbA1C, glycated hemoglobin; BMI, body mass index.

^a^Framingham Risk Assessment Tool is applied to determine 10-year risk of a coronary event (https://www.framinghamheartstudy.org/risk-functions/coronary-heart-disease/hard-10-year-risk.php).
